# The Influence of Loneliness, Social Support and Income on Mental Well-Being

**DOI:** 10.3390/ejihpe15050070

**Published:** 2025-05-06

**Authors:** Eider Egaña-Marcos, Ezequiel Collantes, Alina Diez-Solinska, Garikoiz Azkona

**Affiliations:** 1 Department of Basic Psychological Processes and Their Development, Euskal Herriko Unibertsitatea (UPV/EHU), Tolosa Hiribidea 70, 20018 Donostia, Spain; eider.egana@ehu.eus; 2 Department of Architecture, Euskal Herriko Unibertsitatea (UPV/EHU), Oñati Plaza 2, 20018 Donostia, Spain; ezekiel.collantes@ehu.eus; 3 Department of Health Sciences, Universidad Pública de Navarra (UPNA), Campus de Arrosadía, 31006 Pamplona, Spain; alinaisabel.diez@unavarra.es

**Keywords:** mental well-being, loneliness, social support, income

## Abstract

Mental well-being is a multifaceted concept that reflects emotional stability, psychological resilience and social connectedness. This study examines how demographic factors, perceived loneliness, and social support influence mental well-being in Spain. Participants were surveyed online and provided personal information along with responses to the University of California, Los Angeles (UCLA) Loneliness Scale, the Medical Outcomes Study Social Support Survey (MOS-SSS), and the Warwick–Edinburgh Mental Well-Being Scale (WEMWBS). Our findings support previous research on mental well-being in Spain and again show significant associations between income, loneliness, social support and overall mental health. In particular, perceived loneliness was found to be a strong predictor of mental well-being. Furthermore, income and social support were found to partially mediate the relationship between loneliness and mental well-being. These findings highlight the critical role of social connections and financial stability in promoting mental health. Overall, this research contributes to the growing understanding of the factors influencing mental well-being and provides valuable insights for improving mental health outcomes.

## 1. Introduction

Mental well-being is a complex concept that includes emotional, psychological, and social health. It plays an essential role in an individual’s overall quality of life and significantly influences physical health, functioning, and stress management (Gautam et al., 2024). Mental well-being is not merely the absence of mental disorders but a state of thriving that encompasses emotional balance, psychological resilience, and social connectedness. Emotional health involves the ability to effectively manage and express emotions, while psychological health includes cognitive abilities, self-worth, and a sense of purpose. Social health refers to the ability to build and maintain meaningful relationships and actively engage in community life (Keyes, 2002). Together, these elements contribute to a person’s overall sense of well-being and their capacity to handle life’s challenges.

Over the past decade, the importance of mental well-being has garnered increasing recognition due to its profound impact on both individual and societal health outcomes. As a result, it has become a central topic in public policy, economics, and healthcare, with improving the mental well-being of communities becoming a key societal objective (Ryff, 2014; Steptoe et al., 2015). A wide range of factors, including demographic variables, social context, and access to support systems, influences maintaining mental well-being.

The relationship between demographic characteristics and mental well-being is complex and varies significantly across different populations. For instance, women tend to report lower levels of mental health compared to men (Toribio Caballero et al., 2022). This phenomenon has been attributed to differences in socialization patterns, societal expectations, and the prevalence of gender-based discrimination (Nolen-Hoeksema, 2012). These gender disparities are further influenced by cultural and social contexts, with some studies suggesting that women in more egalitarian societies report better mental health outcomes than those in patriarchal settings (Kiely et al., 2019). Similarly, the relationship between age and well-being is nuanced and varies across different cultural and social contexts (Grossmann et al., 2014; Kim et al., 2021; Ryff, 2014; Saadeh et al., 2020). For example, in some cultures, older adults are highly revered and enjoy robust social support networks, which can significantly enhance their mental well-being. In contrast, in societies where aging is stigmatized, older adults may experience increased feelings of loneliness and social isolation, leading to poorer mental health outcomes (Diener et al., 2013; Donovan & Blazer, 2020; Graham et al., 2011). Previous research in Spain has shown that these demographic factors have little or no effect on mental well-being (Castellví et al., 2014; Forero et al., 2014; Soldevila-Domenech et al., 2021), suggesting that other factors may influence subjective mental well-being.

Recent studies have shown that lower-income working-age adults are particularly susceptible to poor mental health, highlighting the need for targeted interventions to address socioeconomic disparities worldwide (Thomson et al., 2022), and in Spain (Castellví et al., 2014; Macía et al., 2022; Soldevila-Domenech et al., 2021). Individuals with lower incomes and fewer educational opportunities are more likely to experience mental health challenges, including depression and anxiety (Clarke et al., 2000; Keyes et al., 2002). This is often due to the chronic stress associated with financial insecurity, limited access to health care, and exposure to adverse living conditions.

A growing body of research suggests that experiences of loneliness and the availability of social support are key factors influencing mental health outcomes (Wang et al., 2018). Loneliness, often described as the subjective feeling of social isolation, can profoundly affect mental well-being. Unlike objective measures of social isolation, loneliness reflects an individual’s perceived lack of meaningful and satisfying relationships. The mechanisms through which loneliness affects mental well-being are multifaceted. Loneliness can exacerbate existing mental health conditions by amplifying feelings of worthlessness and hopelessness. It can also contribute to the development of new mental health disorders by disrupting sleep patterns, impairing cognitive functioning, and increasing vulnerability to stress (Cacioppo et al., 2010). Furthermore, loneliness often creates a vicious cycle, where individuals who feel lonely are less likely to seek out social interactions, thereby perpetuating their isolation and worsening their mental health. Chronic loneliness is associated with a range of negative psychological outcomes, including depression, anxiety, and increased stress (Cacioppo & Hawkley, 2009; Cacioppo & Patrick, 2008; Mann et al., 2022). These effects are not merely transient; prolonged loneliness can lead to significant and lasting damage to mental health, underscoring the importance of addressing this issue in mental health interventions (Cacioppo et al., 2002; Hawkley et al., 2008; Hawkley et al., 2009).

In contrast to loneliness, social support has consistently been identified as a protective factor for mental health, buffering the harmful effects of stress and loneliness (Cohen & Wills, 1985; Uchino, 2006; Zhang & Dong, 2022). Individuals with strong social support networks report higher levels of life satisfaction, greater emotional stability, and improved coping mechanisms in the face of adversity (Gable & Bedrov, 2022; Thoits, 1995). Social support also plays a crucial role in mitigating the negative effects of loneliness. For example, research has shown that the presence of supportive relationships can partially offset the mental health risks associated with loneliness, highlighting the importance of fostering social connections in mental health interventions (Hutten et al., 2021).

The relationship between mental well-being, loneliness, and social support is complex, influenced by individual differences and contextual factors that affect how social support mitigates loneliness-related distress. While social support can alleviate the negative effects of loneliness, its effectiveness depends on the quality and availability of support networks, as well as individual differences in coping styles and personality traits (Cohen & Wills, 1985; Uchino, 2006). Contextual variables also play a crucial role in shaping the interaction between these factors. In collectivist cultures, where community and family ties are highly valued, social support may be more readily available, providing a stronger buffer against loneliness. In contrast, individualistic societies, which emphasize independence and self-reliance, may leave individuals more vulnerable to the adverse effects of loneliness (Barreto et al., 2021; Ryff, 2014; Schönfeld et al., 2017). This paper explores the relationship between mental well-being, demographic factors, loneliness, and social support in Spain, a country with a moderate score (51 out of 100) on the Hofstede individualism-collectivism scale (Chen-Xia et al., 2023).

## 2. Materials and Methods

Participants were recruited online between July 2023 and January 2025 using a snowball sampling method, where initial respondents were asked to invite others to participate in the survey. The study was limited to individuals aged 18 and older residing in Spain. All participants gave voluntary informed consent before completing the questionnaire, which took approximately 15 min via the Google Drive platform. The study adhered to the ethical guidelines of the Declaration of Helsinki and received approval from the Ethics Committee for Human-Related Research (CEISH) of the University of the Basque Country (UPV/EHU) under protocol number M10/2023/222.

The survey collected participants’ personal information, including gender, sexual orientation, age (categorized as 18–32, 33–45, 46–60, and over 60), sentimental relationship status (yes/no), household composition (living alone or with others), living area (rural or urban), education (primary school, secondary school, vocational training, undergraduate degree, PhD), employment status (retired, studying, both studying and working, unemployed, or working), and annual salary range (less than €28,000, €28,000–€52,000, or over €52,000). In Spain, the average annual salary in 2022 was 26,948.87 euros (INE, 2024). Loneliness was measured using the Spanish version of the UCLA (University of California, Los Angeles) Loneliness Scale (Velarde-Mayol et al., 2016), which consists of 10 items rated on a four-point Likert scale (1 = never; 4 = often), categorized as low (<20), average (20–30), or high (>30). Social support was evaluated using the Spanish version of the Medical Outcomes Study Social Support Survey (MOS-SSS) (Macía et al., 2022), which comprises 19 items rated on a 5-point Likert scale (1 = never, 5 = always). MOS-SSS scores were classified as low (≤38), average (39–57), or high (≥58). Subjective mental well-being was assessed using a 14-item scale rated on a five-point Likert scale (1 = never, 5 = always) (Castellví et al., 2014). WEMWBS scores were categorized as low (≤40), average (41–58), or high (≥59). Participants were grouped into temporal cohorts (quarterly and semi-annually), and their mental well-being scores (WEMWBS) were compared. No statistically significant differences were found between the groups (*p* > 0.05). The lack of significant differences in WEMWBS scores across recruitment periods indicates that recruitment timing did not bias participants’ mental well-being outcomes.

All statistical analyses were conducted using Jamovi (version 2.3.21.0, Sydney, Australia) and GraphPad Prism (version 10.3.1, La Jolla, CA, USA), with a significance level set at *p* < 0.05. Descriptive statistics, including frequencies (%), means ± standard deviations (SD), medians, and ranges, were used to summarize the data. The Shapiro–Wilk test indicated non-parametric distributions for all variables. Consequently, Mann–Whitney U tests were used for comparisons between two groups, while Kruskal–Wallis one-way ANOVA was applied for variables with more than two categories. Effect sizes were calculated using rank biserial correlation (rrb) and Squared-Epsilon coefficient (ε^2^), with interpretations as follows: rrb < 0.3 (small effect), 0.3–0.5 (moderate effect), >0.5 (large effect); ε^2^ 0.01–<0.06 (small effect), 0.06–<0.14 (moderate effect), ≥0.14 (large effect). Associations between variables were analyzed using bivariate Spearman correlation (rho), with interpretations as follows: <0.29 (small effect), 0.30–0.49 (moderate effect), and >0.50 (large effect). Small effect sizes were not considered. Linear regression analyses were performed to determine the independent influence of the variables. Only significant differences between groups are presented in the results, with small effect sizes noted but not emphasized. The statistical analysis excludes items where participants chose not to respond (prefer not to say), as well as groups with only one or two participants (non-binary; asexual; pansexual).

From this first analysis, we observed that three factors (loneliness, social support, and income) can influence mental well-being. They fulfilled the criteria of being statistically significant in the variance analysis, furthermore, they had at least a medium effect size and correlation coefficient. To determine the influence of these three variables independently, we next performed regression and generalized linear model (GLM) analyses.

## 3. Results

### 3.1. Participants’ Information

A total of 506 individuals completed the survey (see Table 1), with the majority identifying as women (71.5%), heterosexual (79.1%), and in the age group 18 to 32 (38.1%). Just over half of the participants were in a relationship (55.3%), and the vast majority lived accompanied at home (83.8%). The majority were working (68.6%) and earned an annual income of less than EUR 28,000 (41.9%). Half of the participants reported experiencing low levels of loneliness (20.1 ± 5.84, median 19, 10–40) and high levels of social support (78.4 ± 14.7, median 82, 26–95).

### 3.2. Mental Well-Being

The WEMWBS (Warwick–Edinburgh Mental Well-being Scale) scores indicate that slightly more than half of the participants (256/50.6%) reported high levels of well-being, while 5.3% (27) scored low (mean = 57.4 ± 9.5; median = 59; range = 14–70). Supplementary Table S1 summarizes categorized WEMWBS results by participants’ personal information.

Significant differences were observed, with a small effect size, regarding household composition (U = 13,783, *p* = 0.003, rbb = 0.207). Participants living accompanied (58.1 ± 8.97, median 59, 21–70) scored higher on the WEMBS compared to those living alone (54.1 ± 11.3, median 54, 14–70). The same trend was observed regarding sentimental status (U = 23,661, *p* < 0.001, rbb = 0.252). Those participants involved in a sentimental relationship score higher (59.3 ± 8.68, median 61.5, 23–70 vs. 55.1 ± 9.98, median 56, 14–70). Similarly, significant differences were observed, with a small effect size, in employment status (X^2^_(4)_ = 14.5, *p* = 0.006, ε^2^ = 0.029). Unemployed individuals (50.8 ± 6.90, median 53, 38–64) scored significantly lower than the university students (56.7 ± 7.98, median 56, 37–70; *p* = 0.032), working university students (56.8 ± 10.9, median 58, 14–70; *p* = 0.042), employed (57.9 ± 9.68, median 60, 21–70; *p* = 0.009) and retirees (59.3 ± 9.03, median 62, 42–70; *p* = 0.036).

**Figure 1:**
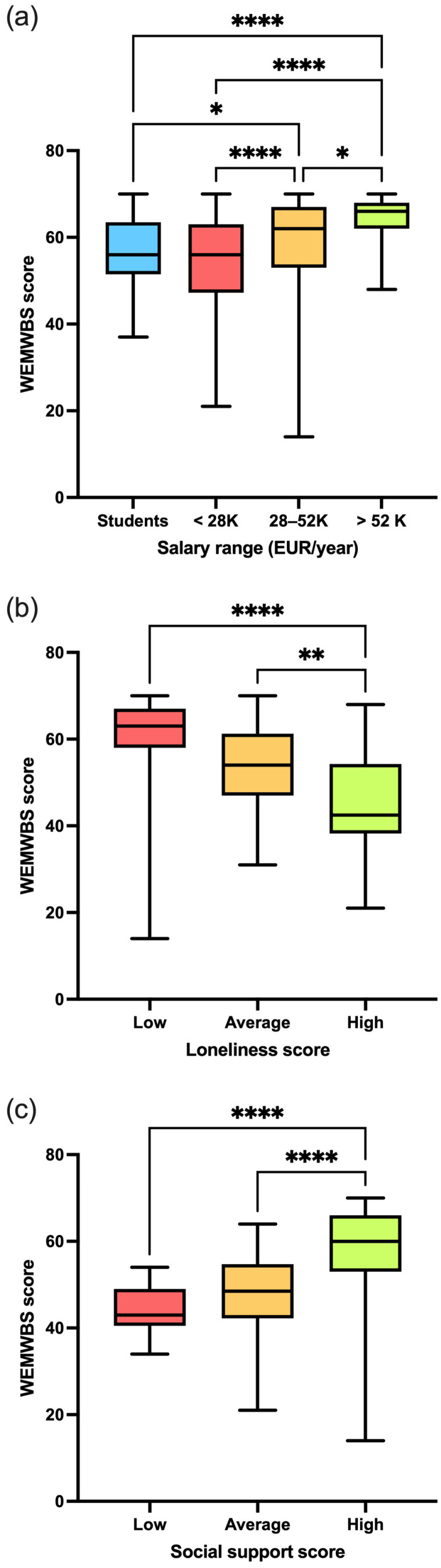
WEMWBS score results by (**a**) salary range, (**b**) UCLA loneliness scale, and (**c**) MOS-SSS scores. Data are presented as group median (min to max). * *p* < 0.05, ** *p* < 0.01, and **** *p* < 0.0001.

Significant differences were found across salary ranges, with a moderate effect size (X^2^_(4)_ = 45.3, *p* < 0.01, ε^2^ = 0.0930). Participants in the lower salary range had the lowest scores (55.0 ± 9.91, median 56, 21–70), followed by university students who were not working (56.5 ± 7.88, median 56, 37–70), those in the intermediate salary range (59.4 ± 9.38, median 62, 14–70), and, lastly, participants in the higher salary range (64.4 ± 5.71, median 66, 48–70) (Figure 1a). The Spearman correlation revealed a moderate positive relationship between salary ranges and WEMBS (0.256, *p* < 0.001). Additionally, significant statistical differences with large effect sizes were observed concerning feelings of loneliness (X^2^_(2)_ = 120, *p* < 0.01, ε^2^ = 0.237). Participants who scored highest on the loneliness scale had the lowest WEMBS scores (45.4 ± 10.8, median 42.5, 21–68), which were significantly lower than those who scored the average (54 ± 8.78, median 54, 31–70) and low (61.6 ± 7.51, median 63, 14–70) ranges on the loneliness scale (Figure 1b). The Spearman correlation revealed a large negative correlation between loneliness scores and WEMBS (−0.593, *p* < 0.001). Finally, significant differences with moderate effect sizes were noted regarding social support (X^2^_(2)_ = 46.3, *p* < 0.01, ε^2^ = 0.0916). Significant differences in WEMBS scores were observed between participants with low (44 ± 6.10, median 43, 34–54) and average (48.4 ± 9.62, median 48.5, 21–64) social support compared to those with high social support (58.4 ± 8.93, median 60, 14–70) (Figure 1c). The Spearman correlation revealed a large positive correlation between social support scores and WEMBS (0.558, *p* < 0.001). No statistical differences were observed for the other variables described in Table 1. Each mean score for these variables is described in Supplementary Table S2.

### 3.3. Linear Regression

Previous analyses have shown that income, loneliness, and social support variables influence WEMBS scores. We performed a regression analysis to determine the influence of each of these variables. The model was adjusted for age, gender, education level, and employment status to account for potential confounding effects. The linear regression model yielded that approximately 29.9% of the variability in total WEMBS is explained by the independent variables (F_(19,464)_ = 11.9; *p* < 0.001; _Adj._R^2^ = 0.299). None of the two-way or three-way interaction terms were statistically significant, suggesting that the effects of loneliness, social support, and income on mental well-being operate independently rather than synergistically (Table 2).

**Figure 2:**
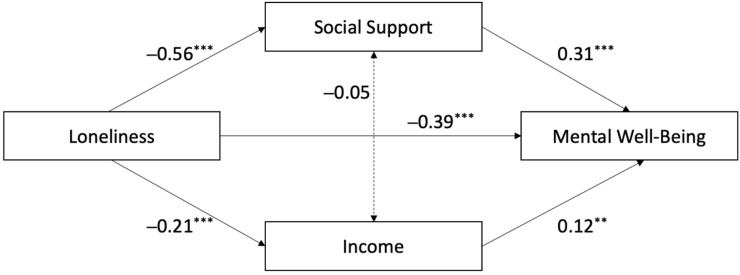
Mediation of the relationship between loneliness and mental well-being, being social support and income mediator. ** *p* < 0.01, *** *p* < 0.001.

To better understand how loneliness influences mental well-being, we conducted a mediation analysis examining the roles of social support and income. The results revealed a partially complementary mediation pattern: while both mediators contributed to explaining the effect of loneliness on mental health, a significant direct effect of loneliness remained (Table 3 and Figure 2). This indicates that loneliness impacts mental well-being not only through reduced social and economic resources but also through other, unmeasured pathways. Among the two mediators, social support showed a stronger indirect effect than income, highlighting its central role in buffering the negative impact of loneliness. Overall, the findings suggest that both social and economic mechanisms partially account for the detrimental effect of loneliness on mental well-being, without fully explaining it.

## 4. Discussion

In our study, perceived mental well-being scores closely align with those reported in the Spanish population (Castellví et al., 2014; Forero et al., 2014; Goñi-Balentziaga & Azkona, 2023; López et al., 2013; Macía et al., 2022; Soldevila-Domenech et al., 2021). These findings suggest that, on average, Spanish citizens may experience higher levels of mental well-being than individuals living in several other European countries, such as Denmark (Koushede et al., 2019), France (Trousselard et al., 2016), Italy (Fazia et al., 2020), and the United Kingdom (Forero et al., 2014; Garrett et al., 2023). As we will discuss below, this could be attributed to differences in social factors.

Consistent with previous studies in Spain, we did not observe gender differences (Castellví et al., 2014; Forero et al., 2014; Soldevila-Domenech et al., 2021), but we were unable to confirm the previously reported negative age-related differences (Castellví et al., 2014; Forero et al., 2014; Soldevila-Domenech et al., 2021). However, it has been noted that the influence of gender and age on mental well-being in the Spanish population is largely attributed to indirect effects, which suggest social inequalities, as other factors mediate their relationship (Soldevila-Domenech et al., 2021). Concerning the other demographic factors examined in our study, unemployment, lack of a partner, or living alone have a small effect on mental well-being, as previously observed in our country (Castellví et al., 2014; Forero et al., 2014; Soldevila-Domenech et al., 2021). Our findings confirm that, in general, demographic factors have little to no effect on mental well-being in the Spanish population, highlighting that other factors, mainly social ones, play a more substantial role in shaping mental well-being. In this regard, and consistent with previous studies, we found that higher-income individuals scored significantly higher on mental well-being measures (Castellví et al., 2014; Soldevila-Domenech et al., 2021). This association could mean that being wealthier may provide individuals with greater access to resources such as psychotherapy, leisure activities, and reduced financial stress, all of which contribute to improved mental well-being, or that better mental well-being allows greater opportunities for income generation. The relationship between income and mental well-being observed in our study provides further insight into the role of socioeconomic factors in mental health.

Our findings confirm that perceived loneliness appears to reduce overall mental well-being, which is consistent with previous research highlighting the detrimental effects of loneliness on mental health (Cacioppo & Hawkley, 2009; Cacioppo et al., 2002; Lyyra et al., 2021; Mushtaq et al., 2014). Conversely, our results show that perceived social support has a positive effect on mental well-being. These results are consistent with the widely held belief that social support greatly enhances happiness (Veenhoven, 2015). Social support acts as a buffer against the detrimental effects of loneliness, significantly enhancing mental well-being. This is consistent with a large body of literature highlighting the protective role of social support in alleviating stress and promoting psychological well-being (Bruss et al., 2024; Cohen & Wills, 1985; Davies et al., 2024; Ginja et al., 2018; Hombrados-Mendieta et al., 2019; Jackman et al., 2023; Loayza-Rivas & Fernández-Castro, 2020; Macía et al., 2022; Soldevila-Domenech et al., 2021). Individuals who perceive themselves as having strong social networks and reliable support systems are better equipped to cope with life’s challenges, enhancing their mental well-being. Thus, the Spanish population may enjoy higher levels of mental well-being compared to the above-mentioned European countries due to higher levels of social support reported (Fernández-Ballesteros, 2002; Salinero-Fort et al., 2011).

One limitation of this study is its reliance on self-reported measures, which may be subject to bias. Additionally, the sample was predominantly female, a trend consistent with many of the studies referenced in the literature. Future research should aim for more balanced gender representation to improve generalizability and provide a broader perspective. Furthermore, the cross-sectional design of this study prevents the establishment of causal relationships between the variables examined. Although instrumental variable (IV) regression is a valuable tool for addressing endogeneity, the current dataset lacks suitable instruments for this approach. Consequently, the findings are framed as correlational and exploratory rather than causal. To better understand the directionality of effects and potential reciprocal relationships, future studies should employ longitudinal or experimental designs.

Despite these limitations, our data confirms the importance of social factors over demographic ones in mental well-being. The interaction between these variables suggests that mental well-being is influenced by a combination of social and economic factors. For example, while higher income may provide individuals with more opportunities to engage in social activities and build supportive networks, the absence of such networks may still lead to feelings of loneliness and reduced well-being.

## 5. Conclusions

Our findings underscore the significant influence of perceived loneliness on mental well-being, with perceived social support and income serving as important mediators. These results emphasize the intricate relationship between individual experiences of loneliness, the availability of social support, financial stability, and overall mental well-being.

## Figures and Tables

**Table 1:** Participant’s personal information.

	*n* (%)
Gender	
Female	362 (71.5%)
Male	140 (27.7%)
Non-binary	1 (0.2%)
Prefer not to say	3 (0.6%)
Sexual orientation	
Asexual	1 (0.2%)
Bisexual	47 (9.3%)
Heterosexual	400 (79.1%)
Homosexual	43 (8.5%)
Pansexual	2 (0.4%)
Prefer not to say	13 (2.6%)
Age	
18–32	193 (38.1%)
33–45	139 (26.9%)
46–60	128 (25.3%)
Over 60	49 (9.7%)
Sentimental relationship	
No	226 (44.7%)
Yes	280 (55.3%)
Household composition	
Living accompanied	424 (83.8%)
Living alone	82 (16.2%)
Living area	
Rural	91 (18%)
Urban	415 (82%)
Education	
Primary school	12 (2.4%)
Secondary school	58 (11.5%)
Vocational training	62 (12.3%)
Undergraduate degree	318 (62.8%)
PhD	56 (11.1%)
Employment status	
Employed	347 (68.6%)
Retired	25 (4.9%)
Unemployed	17 (3.4%)
University student	78 (15.4%)
Working and studying at the University	38 (7.7%)
Salary range (EUR/year)	
Prefer not to say	18 (3.6%)
Students not working	81 (16%)
<28,000	212 (41.9%)
28,000–<52,000	165 (32.6%)
≥52,000	30 (5.9%)
Loneliness	
Low	256 (50.6%)
Average	222 (43.9%)
High	28 (5.5%)
Social support	
Low	9 (1.8%)
Average	44 (8.7%)
High	453 (89.5%)

**Table 2:** Results of the linear regression analysis for mental well-being involving predictor variables of loneliness, social support, and income. Reference level: low, <€28,000, male, 18–32, primary school, employed. * *p* < 0.05, *** *p* < 0.001.

	β	95% CI	t	*p*
Loneliness				
Average	−0.686	−0.84–−0.52	−8.38	<0.001 ***
High	−1.424	−1.78–−1.06	−7.75	<0.001 ***
Social support				
Average	0.097	−0.52–0.70	0.31	0.754
High	0.589	0.02–1.15	2.04	0.025 *
Income				
Students not working	0.085	−0.16–0.33	0.68	0.495
€28,000–€52,000	0.368	0.17–0.56	3.72	<0.001 ***
€52,000	0.680	0.33–1.02	3.88	<0.001 ***
Gender	−0.054	−0.23–0.12	−0.61	0.542
Age				
33–45	−0.071	−0.29–0.15	−0.63	0.527
46–60	−0.047	−0.28–0.18	−0.40	0.689
Over 60	−0.248	−0.55–0.05	−1.60	0.109
Education				
Secondary school	−0.17	−0.71–0.38	−0.61	0.540
Vocational training	−0.11	−0.64–0.42	−0.42	0.670
Undergraduate degree	−0.10	−0.60–0.40	−0.40	0.683
PhD	−0.07	−0.62–0.47	−0.28	0.779
Employment status				
Retired	0.39	−0.06–0.85	1.70	0.088
Unemployed	0.03	−0.44–0.51	0.14	0.889
University student	0.17	−0.38–0.73	0.62	0.532
Working and studying at the University	−0.06	−0.38–0.26	−0.39	0.695
Loneliness * Social Support	0.047	−0.01–0.02-	0.60	0.545
Loneliness * Income	−0.004	−0.82–0.75	−0.08	0.932
Social Support * Income	−0.046	−0.25–0.17	−0.39	0.691
Loneliness * Social Support * Income	0.002	−0.009–0.009	0.06	0.947

**Table 3:** Direct and indirect effects and 95% confidence intervals for the mediation analyses. ** *p* < 0.01, *** *p* < 0.001.

Pathway	β	95% CI	z	*p*
Direct effect				
Loneliness → Mental Well-being	−0.386	−0.77–−0.49	−9.07	<0.001 ***
Indirect effects				
Loneliness → Social Support → Mental Well-being	−0.173	−0.36–−0.20	−6.68	<0.001 ***
Loneliness → Income → Mental Well-being	−0.024	−0.07–−0.02	−2.70	0.007 **
Total	−0.582	−1.06–0.83	−16.09	<0.001 ***

## Data Availability

The study data will be made available upon reasonable request to the corresponding author.

## Supplementary Materials

The following supporting information can be downloaded at: https://www.mdpi.com/article/10.3390/ejihpe15050070/s1, Table S1: Categorized WEMWBS results based on participants’ personal information; Table S2: WEMWBS score results.

## Abbreviations

The following abbreviations are used in this manuscript:

MOS-SSS	Medical Outcomes Study Social Support Survey
SD	Standard Deviation
UCLA	University of California, Los Angeles
WEMWBS	Warwick–Edinburgh Mental Well-Being Scale
