# Supplementary material for: The Influence of Loneliness, Social Support and Income on Mental Well-Being
Source: Eur J Investig Health Psychol Educ. 2025 May 6;15(5):70. doi: 10.3390/ejihpe15050070 (PMC12109767; doi:10.3390/ejihpe15050070)
Supplement: Supplementary file 1 [file ejihpe-15-00070-s001.zip › ejihpe-3584562-supplementary.pdf]

Supplementary Table S1. Categorized WEMWBS results based on participants' personal information.

	Low	Average	High
	n (%)		
**Gender**			
Female	19 (3.8%)	170 (33.9%)	175 (34.9%)
Male	7 (1.4%)	53 (10.6%)	78 (15.5%)
Non-binary	0 (0.0%)	0 (0.0%)	1 (0.2%)
**Sexual orientation**			
Asexual	0 (0.0%)	1 (0.2%)	0 (0.0%)
Bisexual	3 (0.6%)	27 (5.5 %)	17 (3.5%)
Heterosexual	18 (3.7%)	175 (35.6%)	207 (42.1%)
Homosexual	4 (0.8%)	14 (2.8%)	25 (5.1%)
Pansexual	0 (0.0%)	0 (0.0%)	2 (0.4%)
**Age**			
18-32	9 (1.8%)	86 (17.0%)	98 (19.4%)
33-45	9 (1.8%)	57 (11.3%)	70 (13.8%)
46-60	6 (1.2%)	42 (8.3%)	80 15.8%)
Over 60	1 (0.2%)	23 (4.5%)	25 (4.9%)
**Sentimental relationship**			
No	18 (3.6%)	120 (23.7%)	88 (17.4%)
Yes	9 (1.8%)	104 (20.6%)	167 (33%)
**Household composition**			
Live accompanied	16 (3.2%)	188 (37.2%)	220 (43.5%)
Living alone	11 (2.2%)	36 (7.1%)	35 (6.9%)
**Living area**			
Rural	5 (1.0%)	43 (8.5%)	43 (8.5%)
Urban	22 (4.3%)	181 (35.8%)	212 (41.9%)
**Employment status**			
Employed	20 (4%)	136 (26.9%)	191 (37.7 %)
Retired	0 (0%)	10 (2%)	15 (3%)
Unemployed	2 (0.4%)	14 (2.8%)	1 (0.2%)
University student	2 (0.4%)	45 (8.9%)	31 (6.1%)
Working and studying at the University	3 (0.6%)	19 (3.8%)	17 (3.4%)
**Salary range** (euros/year)			
Prefer not to say	0 (0.0%)	9 (1.8%)	9 (1.8%)
Students not working	2 (0.4%)	49 (10%)	30 (6.1%)
< 28,000	19 (3.9%)	104 (21.3%)	89 (18.2%)
28,000 - < 52,000	6 (1.2%)	57 (11.7%)	102 (20.9%)
≥ 52,000	0 (0.0%)	4 (0.8%)	26 (5.3%)

Supplementary Table S2. WEMWBS score results.

	Mean	SD	Median	Range
**Gender**				
Female	57	9.18	58	14-70
Male	58.7	9.43	61	30-70
Non-binary	65	NaN	65	NaN
**Sexual orientation**				
Asexual	45.0	NaN	45	NaN
Bisexual	55.3	9.72	56	37-70
Heterosexual	57.7	9.37	59	14-70
Homosexual	58.1	9.71	61	37-70
Pansexual	65	1.41	65	64-66
**Age**				
18-32	56.8	9	58	21-70
33-45	56.9	10.1	58.5	31-70
46-60	58.7	9.93	61.5	14-70
Over 60	57.9	8.41	58	38-70
**Living area**				
Rural	57.2	9.10	58	33-70
Urban	57.5	9.60	59	14-70
